# Epidemiological Trends of Traumatic Brain Injury Identified in the Emergency Department in a Publicly-Insured Population, 2002-2010

**DOI:** 10.1371/journal.pone.0145469

**Published:** 2016-01-13

**Authors:** Terence S. Fu, Ruwei Jing, Wayne W. Fu, Michael D. Cusimano

**Affiliations:** 1 Division of Neurosurgery, Department of Surgery, St. Michael’s Hospital; Injury Prevention Research Office, Li Ka Shing Knowledge Institute, Keenan Research Centre; University of Toronto, Toronto, ON, Canada; 2 Dalla Lana School of Public Health, University of Toronto, Toronto, Canada; University of Pittsburgh, UNITED STATES

## Abstract

**Objectives:**

To examine epidemiological trends of Traumatic Brain Injury (TBI) treated in the Emergency Department (ED), identify demographic groups at risk of TBI, and determine the factors associated with hospitalization following an ED visit for TBI.

**Methods:**

A province-wide database was used to identify all ED visits for TBI in Ontario, Canada between April 2002 and March 2010. Trends were analyzed using linear regression, and predictors of hospital admission were evaluated using logistic regression.

**Results:**

There were 986,194 ED visits for TBI over the eight-year study period, resulting in 49,290 hospitalizations and 1,072 deaths. The age- and sex-adjusted rate of TBI decreased by 3%, from 1,013.9 per 100,000 (95% CI 1,008.3–1,010.6) to 979.1 per 100,000 (95% CI 973.7–984.4; p = 0.11). We found trends towards increasing age, comorbidity level, length of stay, and ambulatory transport use. Children and young adults (ages 5–24) sustained peak rates of motor vehicle crash (MVC) and bicyclist-related TBI, but also experienced the greatest decline in these rates (p = 0.003 and p = 0.005). In contrast, peak rates of fall-related TBI occurred among the youngest (ages 0–4) and oldest (ages 85+) segments of the population, but rates remained stable over time (p = 0.52 and 0.54). The 5–24 age group also sustained the highest rates of sports-related TBI but rates remained stable (p = 0.80). On multivariate analysis, the odds of hospital admission decreased by 1% for each year over the study period (OR = 0.991, 95% CI = 0.987–0.995). Increasing age and comorbidity, male sex, and ambulatory transport were significant predictors of hospital admission.

**Conclusions:**

ED visits for TBI are involving older populations with increasingly complex comorbidities. While TBI rates are either stable or declining among vulnerable groups such as young drivers, youth athletes, and the elderly, these populations remain key targets for focused injury prevention and surveillance. Clinicians in the ED setting should be cognizant of factors associated with hospitalization following TBI.

**Level of Evidence:**

III.

**Study Design:**

Cross-sectional.

## Introduction

Traumatic brain injury (TBI) is a major cause of traumatic death and disability globally, and therefore requires ongoing prevention and surveillance. In the United States, 1.7 million TBIs occur annually, and an estimated 2% of the population lives with a TBI-related disability [1]. TBI is frequently associated with long-term neuropsychiatric sequelae, including cognitive dysfunction, depression, and anxiety [2, 3].

Approximately 80–92% of TBIs are treated in the Emergency Department (ED) [1, 4]. Each year, there are 1.3 million ED visits for TBI in the US, resulting in $5.8 billion (USD) in direct healthcare costs alone [1, 5]. Furthermore, studies have shown a recent surge in ED visits related to TBI although the cause of this rise is unclear [1, 6, 7].

Monitoring long-term epidemiological patterns of TBI-related ED visits and identifying clinical predictors of hospital admission are important for developing targeted health policy and injury prevention measures. Yet, despite the growing prevalence and economic burden of TBI treated in the ED, there is limited research examining temporal trends in this critical setting. We aim to build on previous studies that lack long-term data [6, 8], focus on specific subpopulations [9–11], or do not analyze trends in detail [1, 3, 7, 12]. Furthermore, to our knowledge, there is only one study that has examined a range of clinical predictors of hospital admission following TBI. McCarthy et al. [13] identified factors influencing hospitalization for pediatric TBI in Maryland, USA using 1998 statewide data. However, this study is outdated and based on a small patient population, and may not be generalizable to all age groups.

The present study examines recent trends in ED visits and admissions associated with TBI over an eight-year period from 2002/03 to 2009/10 in Ontario, Canada’s most populous province. We examine injury rates across multiple strata including age, sex, and mechanism of injury, and identify factors influencing hospital admission using a province-wide database that is mandatory for all EDs and ambulatory care centers in Ontario. The aims of this study are four-fold: (1) to describe epidemiological trends for TBI-related ED visits, (2) identify key demographics at risk of TBI, (3) elucidate the predictors of hospitalization following an ED visit for TBI, and (4) assess the implications of these findings on health policy and injury prevention.

## Methods

### Study design and setting

We conducted a population-based descriptive epidemiological study of TBI visits to Ontario EDs over an eight-year period. Incidence data was obtained from the National Ambulatory Care Resource System (NACRS), a mandatory reporting database for all EDs and ambulatory care centers in Ontario. NACRS collects information on patient identifiers (i.e. unique ID, health card number, postal code) and demographics (gender, birth date, education level), up to ten International Classification of Diseases, Tenth Revision (ICD-10) diagnosis codes, external causes of injury codes, ambulatory transport, and visit disposition. A recent re-abstraction study reported high agreement rates in diagnosis coding between NACRS and 7,500 charts from fifteen Ontario EDs [14]. Approval for this study was obtained from the Research Ethics Board at St. Michael’s Hospital. Informed consent was waived as this study was based on de-identified administrative data.

### Study population

The study population included all ED visits between April 1, 2002 and March 31, 2010 that contained an ICD-10 code corresponding to TBI in any diagnosis field. TBI was defined using the following ICD-10 codes: open wound of head [S01(.7,.8,.9)], fracture of skull and facial bones [S02(.0,.1,.7-.9)], intracranial injury (S06.0-S06.9), crushing injury of head [S07(.1,.8,.9)], unspecified injury of head (S09.7-S09.9), injuries involving head with neck (T02.0,T04.0,T06.0), and sequelae of injuries of head [T90(.2,.5,.8,.9)]. The Centers for Disease Control and Prevention (CDC) [1] includes additional ICD-10 codes in their definition of TBI mortality. We chose a more conservative set of codes to capture TBI morbidity based on previous studies [15, 16] and the author’s (M.C.) 30+ years of clinical experience. Patients who registered but left before treatment were excluded from this study. Mechanisms of injury were defined using the CDC’s External Cause of Injury Matrix [17] and collapsed into several main categories: motor vehicle collisions, falls, struck by/against, sports injuries, cyclist-related injuries, and other causes.

ICD-10 codes were used to calculate the Charlson Comorbidity Index (CCI) for each patient using a validated ICD-10 coding algorithm [18]. The CCI is a widely-used indicator of disease burden which identifies 19 clinical conditions that are significant predictors of mortality, including congestive heart failure, liver disease, and renal disease [19]. All diagnosis fields were searched for an ICD-10 code corresponding to any of these conditions, and the total score was calculated for each patient. Use of ambulatory transport was designated as a proxy for injury severity, as traditional measures of severity (e.g. Abbreviated Injury Scale, Injury Severity Score, Glasgow Coma Scale) were either not reported or missing in most cases.

### Measurements and data analysis

Descriptive statistics were used to describe the study cohort. Age- and sex-specific rates were calculated using population data from Statistics Canada, and reported with 95% confidence intervals (CI). Linear regression was used to test for significant trends in TBI rates over the eight-year study period, and to evaluate trends in continuous variables such as age, CCI, and length of stay (LOS). It should be noted that the NACRS definition of ED LOS changed in fiscal year 2007/08 with the introduction of clinical decision units in Ontario; therefore, LOS cannot be compared across the entire study period. As such, data on LOS were only assessed for the five-year period prior to 2007/08 [20].

A Chi-square test was used to compare characteristics of admitted vs. non-admitted patients sustaining a non-fatal TBI. Logistic regression was then used to model binary outcomes for hospital admission or transfer to another acute care facility (vs. discharged home). Adjusted and unadjusted odds ratios (OR) were calculated with corresponding 95% CIs. Multicollinearity was assessed with a variance inflation factor over 4. All statistical analyses were performed using SAS 9.4 (SAS Institute, Inc., Cary, NC, USA). A p-value less than 0.05 was considered statistically significant.

## Results

Between 2002/03 and 2009/10, there were 986,194 ED visits for TBI, resulting in 1,072 deaths. The annual number of ED visits for TBI increased 4% from 122,620 to 127,255 over the eight-year study period (Table 1). However, the overall rate of these visits decreased 3% from 1,013.9 (95% CI 1,008.3–1,010.6) to 979.1 per 100,000 (95% CI 937.7–984.4), although no significant linear trend was detected (p = 0.11).

**Table 1:** Characteristics of TBI visits to emergency departments, 2002/03 to 2009/10.

	Incidence		Rate (95% CI)*		Percent Change	Slope of Regression	p-value^†^
	2002	2009	2002	2009			
**Overall**	122,620	127,255	1,014 (1,008–1,020)	979.1 (973.7–984.4)	-3%	-29.9	0.11
**Gender**							
Male	78,009	75,644	1,305 (1,296–1,314)	1,184 (1,175–1,192)	-9%	-45.0	0.04
Female	44,611	51,611	729.3 (722.6–736.1)	781.1 (774.4–787.8)	7%	-14.8	0.58
**Age**							
0–4	23,580	23,408	3,396 (3,353–3,438)	3,317 (3,275–3,358)	-2%	-80.5	0.20
5–14	26,661	23,045	1,649 (1,629–1,668)	1,519 (1,500–1,539)	-8%	-55.4	0.05
15–24	22,609	23,694	1,393 (1,375–1,411)	1,332 (1,315–1,349)	-4%	-47.4	0.09
25–34	12,484	11,698	729.6 (716.8–742.3)	680.0 (667.7–692.3)	-7%	-22.9	0.08
35–44	11,457	9,893	554.3 (544.2–564.5)	522.0 (511.7–532.2)	-6%	-19.0	0.07
45–54	7,921	10,084	462.8 (452.6–472.9)	487.9 (478.4–497.4)	5%	-8.6	0.39
55–64	5,034	7,157	436.5 (424.5–448.6)	466.7 (455.9–477.5)	7%	-7.0	0.57
65–74	4,309	5,540	516.8 (501.4–532.2)	586.0 (570.6–601.4)	13%	-5.6	0.90
75–84	5,415	7,353	1,022 (995–1,049)	1,203 (1176–1,231)	18%	-11.4	0.89
85+	3,150	5,383	2,064 (1,993–2,135)	2,403 (2,339–2,466)	16%	-18.0	0.92
**Mechanism of injury**^‡^							
Fall	51,297	60,577	424.2 (420.5–427.8)	466.1 (462.4–469.8)	10%	-4.0	0.99
Struck	43,903	45,860	363.0 (359.7–366.4)	352.8 (349.6–356.1)	-3%	-11.4	0.12
MVC	15,242	13,060	126.0 (124.0–128.0)	100.5 (98.8–102.2)	-20%	-6.3	0.004
Other ^§^	12,178	7,758	100.7 (98.9–102.5)	059.7 (58.4–061.0)	-41%	-8.2	0.002
Sports	10,810	12,079	89.4 (87.7–91.1)	92.9 (91.3–94.6)	4%	-2.2	0.35
Bicyclist	3,958	3,924	32.7 (31.7–33.8)	30.2 (29.3–31.1)	-8%	-1.4	0.06
**Charlson Comorbidity Index**							
0–1	107,462	105,091	888.6 (883.3–893.9)	808.5 (803.7–813.4)	-9%	-31.9	0.04
2–3	9,161	11,798	75.8 (74.2–77.3)	90.8 (89.1–92.4)	20%	-0.5	0.69
4+	5,997	10,366	49.6 (48.3–50.8)	79.8 (78.2–81.3)	61%	2.5	0.004
**Visit Disposition**							
Home	114,329	115,227	945.4 (939.9–950.9)	886.5 (881.4–891.6)	-6%	-32.5	0.07
Admitted to reporting hospital	5,985	6,177	49.5 (48.2–50.7)	47.5 (46.3–48.7)	-4%	-1.5	0.07
Transfer, other acute care	1,697	2,164	14.0 (13.4–14.7)	16.7 (16.0–17.4)	19%	0.0	0.56
Transfer, non-acute care	176	164	1.5 (1.2–1.7)	1.3 (1.1–1.5)	-13%	-0.1	0.11
Died	156	134	1.3 (1.1–1.5)	1.0 (0.9–1.2)	-20%	-0.1	0.01
Other ^||^	277	3,389	2.3 (2.0–2.6)	26.1 (25.2–27.0)	1038%	4.3	0.01
**Ambulance transport**							
Air	1,137	261	9.4 (8.9–10.0)	2.0 (1.8–2.3)	-79%	-0.8	0.18
Ground	23,167	29,019	191.6 (189.1–194.0)	223.3 (220.7–225.8)	17%	0.0	0.89
Combination (air and ground)	155	212	1.3 (1.1–1.5)	1.6 (1.4–1.9)	27%	-1.5	0.37
None	98,161	97,763	813.4 (810.0–816.8)	752.2 (747.5–756.9)	-8%	-27.3	0.30
**Length of Stay (hours)**^¶^							
0–1	30,308	28,941	239.4 (236.7–242.1)	222.7 (220.1–225.2)	-7%	45.9	0.01
1–2	27,673	30,040	218.6 (216.0–221.1)	231.1 (228.5–233.7)	6%	44.8	0.004
2–3	19,267	23,001	152.2 (150.0–154.3)	177.0 (174.7–179.3)	16%	33.2	0.003
3–5	20,003	25,258	158.0 (155.8–160.2)	194.3 (191.9–196.7)	23%	35.5	0.002
5+	13,803	19,467	109.0 (107.2–110.8)	149.8 (147.7–151.9)	37%	26.1	0.001
Missing	1,728	548	13.7 (13.0–14.3)	4.2 (3.9–4.6)	-100%	-215.4	0.01

Abbreviations: TBI, traumatic brain injury; CI, confidence interval.

* Rate per 100,000; calculated using population data from Statistics Canada.

^†^ Tested for trend significance using linear regression analysis.

^‡^ Fall, struck, MVC, and other injuries represent 100% of injuries. Sports and bicyclist injuries are reported separately.

^§^ Cut/pierce; drowning/submersion; firearm; machinery; pedal cyclist, pedestrian, or transport (not motor vehicle crash-related); natural/environmental; other specified; unspecified; and adverse effects.

^||^ Transferred to other healthcare facility (e.g. hospice, palliative care, outpatient clinic), signed out against medical advice, unknown disposition.

^¶^ Data on length of stay (LOS) were presented for the five-year period prior to 2007/08, as the NACRS definition of LOS changed in 2007/08 with the introduction of clinical decision units in Ontario.

The most common presenting complaints for TBI were “unspecified injuries” (49%), open head wounds (37%), and intracranial injuries (12%). The majority of patients (92%) were discharged home, while 5% were admitted to hospital, 2% were transferred to an acute care facility, and the remainder of patients were transferred to another type of non-acute healthcare facility (e.g. hospice, outpatient clinic).

### Age- and sex-related trends

TBI rates for males were consistently 60–80% higher than those for females. Over the study period, the overall rate among males declined 9% from 1,305.3 to 1,183.8 per 100,000, while the overall rate among females increased 7% from 729.3 to 781.1 per 100,000 (Table 1).

**Fig 1:**
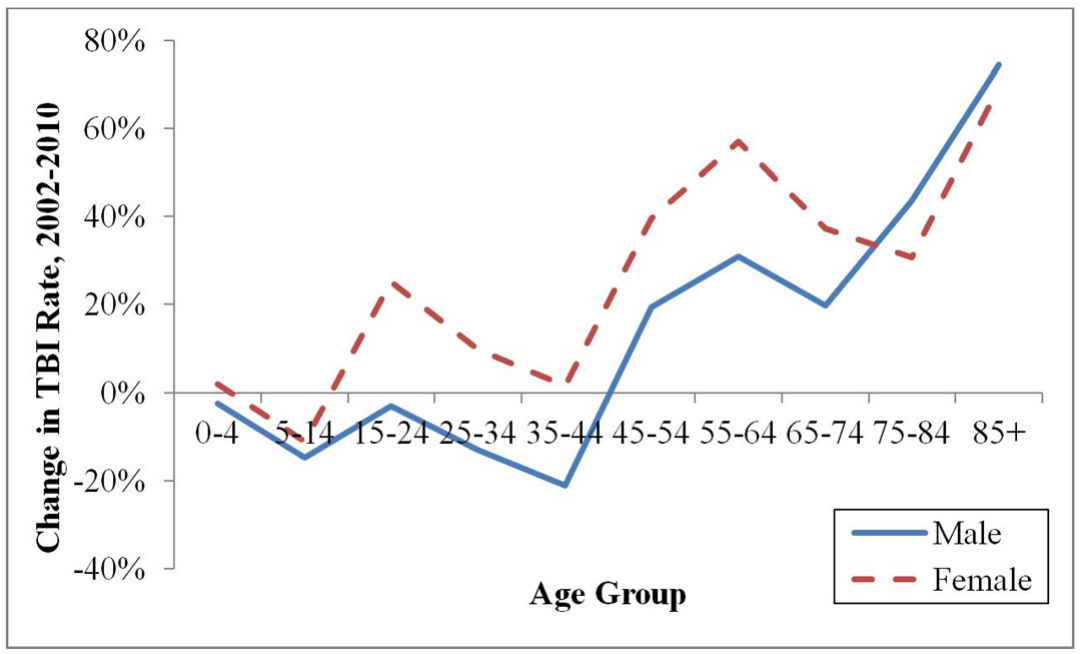
Percent change in rate of emergency department visits for traumatic brain injury (TBI) visits by age group and sex, 2002/03 to 2009/10.

These overall trends conceal major age- and sex-related differences depicted in Fig 1. Rates among males declined for all age groups under 65, but increased 17% among those ages 65 and older. In contrast, rates increased for all female age groups except for the 5–14 age group. However, among both sexes, the greatest and most significant increases occurred among those ages 85 and older (74% among males, p = 0.002; 69% among females, p = 0.004). Together, these trends resulted in significant increases over time in the mean age from 26.4 to 30.0 (p<0.001) and median age from 19.0 to 21.0 (p = 0.002) among TBI patients seeking ED treatment.

### Time- and age-related trends by mechanism of injury

Falls, struck by/against, and motor vehicle collisions (MVCs) were the most common mechanisms of TBI, representing 45%, 36%, and 11% of ED visits, respectively (Table 2). Sports- and bicyclist-related injuries accounted for 18% and 6% of TBIs, respectively.

**Table 2:** Comparison of admitted and non-admitted TBI patients presenting to emergency departments.

	Non-admitted, n (%)	Admitted, n (%)	p-value
**Overall**	935,832	49,290	
**Age** median (IQR)	19 (35)	48 (54)	<0.0001
**Gender**			
Female	354,721 (38%)	18,341 (37%)	0.0004
Male	581,111 (62%)	30,949 (63%)	
**Mechanism of injury**			
Fall	413,159 (44%)	26,132 (53%)	<.0001
Struck	349,858 (37%)	6,210 (13%)	
MVC	94,917 (10%)	13,516 (27%)	
Other	77,899 (8%)	3,432 (7%)	
Sports	88,681 (9%)	1,261 (3%)	<.0001
Bicyclist	29,921 (3%)	1,879 (4%)	<.0001
**Charlson Comorbidity Index** mean (SD)	0.49 (1.16)	1.5 (1.83)	<.0001
**Ambulance transport**			
Air	1,227 (0.1%)	1,103 (2%)	<.0001
Ground	455 (0.05%)	0,840 (2%)	
Combination	188,250 (20%)	32,670 (66%)	
None	745,900 (80%)	12,833 (26%)	
**Length of stay in hours**, median (IQR)	1.9(2.6)	4.2(6.3)	<.0001

Abbreviations: TBI, traumatic brain injury; IQR, interquartile range; SD, standard deviation.

**Fig 2:**
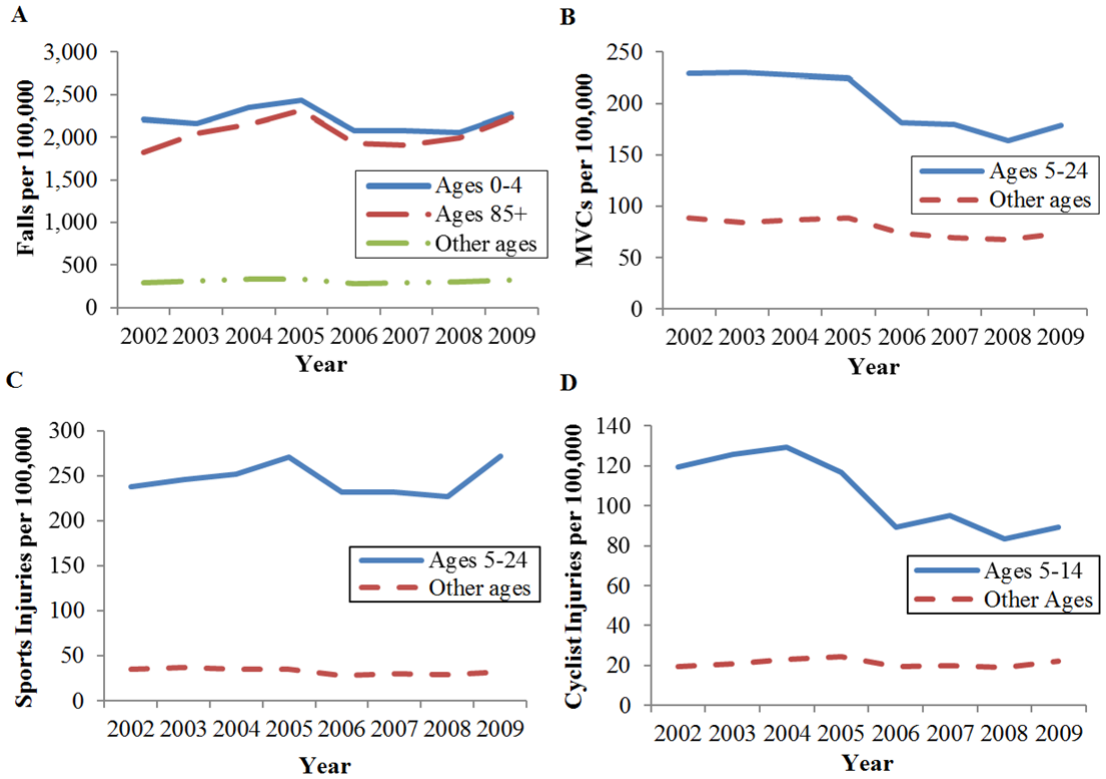
Rate of emergency department visits by age group for traumatic brain injury caused by (A) falls, (B) motor vehicle crashes (MVCs), (C) sports injuries, and (D) cyclist injuries.

The rate of fall-related TBIs remained stable between 2002 and 2010 with an average rate of 438.6 per 100,000. Young children (ages 0–4) and elderly adults (ages 85+) were most vulnerable to sustaining a fall-related TBI, with seven-fold higher rates among young children and elderly adults compared to other age groups (Fig 2A). However, no significant trends were detected in fall rates for any age group.

Conversely, the rate of MVCs decreased among all age groups, with an overall 20% decrease over the study period and a significant decreasing trend (p = 0.004). Peak rates of MVCs occurred among children/adolescents (5–14) and young adults (ages 15–24). Together, these vulnerable age groups sustained 2.5-fold higher MVC rates compared to all other age groups (Fig 2B). However, the 5–24 age group also experienced the greatest decline in MVC rates over the study period. MVC rates among children/adolescents decreased 26% from 196.0 to 144.8 per 100,000 (p = 0.003), and rates among young adults decreased 21% from 262.0 to 207.4 per 100,000 (p = 0.003).

Sports-related TBIs demonstrated a similar age distribution, with peak incidence and rates occurring among those ages 5–24. This age cohort had a seven-fold higher rate of sports-related TBI in comparison to all other age groups. Rates remained stable at 250.1 and 242.2 per 100,000 for those ages 5–14 and 15–24, respectively (p = 0.64 and p = 0.95; Fig 2C). Meanwhile, the 5–14 age group suffered a disproportionate number of bicyclist-related TBIs, and also experienced a five-fold higher rate of injury compared to other ages (Fig 2D). However, this age group also showed the greatest decline in bicyclist-related injury rates, which decreased 25% from 119.5 to 89.2 per 100,000 (p = 0.005).

### Trends in ambulatory transport, comorbidity, and length of stay

Over the study period, TBI patients presented to EDs with increasing ambulatory transport use, comorbidity level, and length of stay. TBI rates among patients with the highest comorbidity level (CCI over 4) increased 61% over the study period (p = 0.004), with a concomitant 9% decrease among patients with the fewest comorbidities (CCI 0–1; p = 0.04). On average, the CCI increased 47% from 0.45 to 0.66 over the eight-year study period (p<0.001). Additionally, median length of stay (LOS) increased 16% from 1.9 to 2.2 between 2002/03 and 2006/07 (p = 0.002). The rate of TBI with LOS less than one hour decreased significantly (p = 0.01), while rates among longer LOS categories increased significantly during this period. Ambulance transport was used as a rough proxy for injury severity, with more severely injured patients more likely to require air and/or ground transport. Over the eight-year study period, the rate of transported patients increased 12% (p = 0.08) while the rate of non-transported patients decreased 8% (p = 0.30).

### Trends in hospital admission patterns

The number of hospital admissions for TBIs presenting to the ED increased 3% over the study period from 5,985 to 6,177 per year, but the rate of hospital admission decreased 4% from 49.5 to 47.5 per 100,000 (p = 0.07; Table 1). Characteristics of admitted and non-admitted (non-fatal) patients were compared (Table 2). Compared to non-admitted patients, hospitalized patients were older (48 vs. 19 years, p<0.0001) and more comorbid (CCI 1.5 vs. 0.49, p<0.0001), and experienced longer ED visits (4.2 vs. 1.9 hours, p<0.0001). Additionally, a greater proportion of admitted patients were male (63% vs. 62%, p = 0.0004), required ambulatory transport (74% vs. 20%, p<0.0001), and involved a fall (53% vs. 44%) or MVC (27% vs. 10%).

On univariable analysis, increasing age, comorbidity level, and length of stay were clearly associated with higher odds of admission (Table 3). Male sex, MVCs, falls, and bicyclist-related injuries were also associated with increased likelihood of admission. Patients requiring any form of ambulatory transport also had considerably increased odds of admission. The odds of hospitalization increased slightly by 0.5% each year of the study period.

**Table 3:** Predictors of hospital admission following TBI visit to the emergency department.

	Odds Ratio (95% CI)	p-value	Adj. Odds Ratio (95% CI)	Adj. p-value
**Age (vs. 0–4)**				
0–4	1.00		1.00	
5–14	1.34(1.29,1.40)	<.0001	1.20(1.15,1.26)	<.0001
15–24	2.20(2.12,2.28)	<.0001	1.33(1.28,1.39)	<.0001
25–34	2.47(2.37,2.57)	<.0001	1.42(1.35,1.49)	<.0001
35–44	3.08(2.95,3.20)	<.0001	1.61(1.53,1.68)	<.0001
45–54	4.06(3.90,4.23)	<.0001	1.93(1.85,2.02)	<.0001
55–64	5.44(5.22,5.67)	<.0001	2.13(2.02,2.25)	<.0001
65–74	8.23(7.90,8.58)	<.0001	2.62(2.43,2.82)	<.0001
75–84	9.96(9.59,10.34)	<.0001	2.44(2.26,2.63)	<.0001
85+	10.58(10.16,11.02)	<.0001	1.94(1.78,2.11)	<.0001
**Gender**				
Female	1.00		1.00	
Male	1.03(1.01,1.05)	0.0004	1.53(1.50,1.56)	<.0001
**Mechanism of injury**				
Other	1.00		1.00	
Struck	0.40(0.39,0.42)	<.0001	0.50(0.48,0.52)	<.0001
Fall	1.44(1.39,1.48)	<.0001	1.00(0.97,1.04)	0.93
MVC	3.23(3.12,3.34)	<.0001	2.02(1.95,2.10)	<.0001
**Sports (vs. non-sports)**	0.25(0.24,0.26)	<.0001	0.94(0.89,0.99)	0.04
**Bicyclist (vs. non-bicyclist)**	1.20(1.15,1.25)	<.0001	0.62(0.59,0.66)	<.0001
**Charlson Comorbidity Index**				
0–1	1.00			
2–3	3.63(3.55,3.71)	<.0001	1.33(1.26,1.41)	<0.001
4+	5.35(5.23,5.46)	<.0001	1.79(1.66,1.92)	<.0001
**Ambulance transport**				
None	1.00			
Ground	10.1(9.9,10.3)	0.0028	5.99(5.87,6.11)	<.0001
Air	52.2(48.2,56.6)	<.0001	33.4(30.7,36.3)	<.0001
Combination	107.2(95.5,120.3)	<.0001	70.9(62.8,80.0)	<.0001
**Year**	1.005(1.001,1.008)	<.0001	0.991(0.987,0.995)	<.0001

Abbreviations: TBI, traumatic brain injury.

Model performance was assessed, with area under the receiver operating characteristic curve (AUROC) = 0.8291; Wald χ2 = 76,825 (p<0.0001).

On multivariable analysis, increasing age remained predictive of hospital admission, but the 65–74 age group experienced the greatest odds of admission after controlling for relevant factors (OR = 2.62, p<0.001, 95% CI = 2.43–2.82). Males also had a 53% higher risk of admission compared to females (p<0.0001, 95% CI = 1.50–1.56). In terms of mechanism of injury, only MVCs were associated with increased odds of hospital admission (OR = 2.02, 1.95–2.10, p<0.0001). Comorbidity level was also predictive of hospital admission, with highly comorbid patients (CCI>4) 79% more likely to be admitted compared to the least comorbid group (p<0.0001, 95% CI = 1.66–1.92). Ambulance transport remained the most important predictor of hospital admission. Patients requiring ground transport were six times more likely to be admitted than those not requiring transport (p<0.0001, 95% CI = 5.87–6.11); those requiring air transport were 33 times more likely to be admitted (p<0.0001, 95% CI = 30.7–36.3); and those requiring a combination of transport modalities were nearly 71-fold more likely to be admitted (p<0.0001, 95% CI = 62.8–80.0). After controlling for relevant factors, the odds of hospitalization decreased by approximately 1% per year over the study period (p<0.0001; OR = 0.991, 95% CI = 0.987–0.995).

## Discussion

Examining long-term epidemiological trends across a range of strata is crucial for targeting and evaluating health policy and injury prevention measures, and reliable baseline data is needed to accurately assess the healthcare burden of TBI. Ongoing surveillance of TBI patterns is of particular importance in the ambulatory care context, as the majority of these injuries are treated in these settings and recent studies have indicated a surge in ED visits associated with TBI. The present study describes trends in TBI rates and hospital admissions in Ontario over an eight-year period from 2002/03 to 2009/10. We provide a critical update of the ED burden associated with TBI and highlight demographic groups at risk of sustaining a TBI requiring ED attention.

Comparisons with findings from the literature are difficult as rates vary widely depending on the time period, geographical location, and definition of TBI, and there are no other longitudinal studies of ED visits for TBI over a comparable period in any publicly-insured population. Recent US studies have found increasing trends in TBI-related ED visits, with rates increasing by 12% to 84% over various time periods[1, 3, 6, 7]. To our knowledge, there is only one report summarizing trends in TBI visits to EDs in Canada. A 2007 report [12] by the Canadian Institute for Health Information (CIHI) found that the number of ED and urgent care center visits for TBI in Canada decreased 2% from 82,831 to 80,970 between 2002 and 2006. Based on population estimates from Statistics Canada, this represents a 6% decrease in the overall rate of ambulatory care visits for TBI. In contrast, our study did not detect a significant linear trend in overall TBI rates during a comparable time period between 2002 and 2009 (p = 0.11). It is important, however, to recognize that these overall rates conceal major age-, sex- and mechanism-specific trends. Thus, our study highlights the need for enhanced surveillance of detailed TBI trends across important strata.

Patterns of TBI by age, sex, and mechanism of injury identified in this study are consistent with those reported in the literature. TBIs presenting to ED are known to occur disproportionately among males and those at extreme ends of the age spectrum [3, 6, 8]. Falls [1, 6, 8, 10, 11, 21, 22], MVCs [1, 6, 10, 22–24], and sports and bicyclist injuries [9, 10, 25, 26] are also common mechanisms of TBI treated in the ED. Previous studies have shown that peak rates of fall-related TBI occur among young children (0–4) and elderly adults (85+) [1, 10, 11, 21]. Additionally, the Centers for Disease Control and Prevention [1] reported a 62% increase in fall-related TBI rates among young children and a 46% increase in rates among elderly adults in the US between 2002 and 2006. Our study found a modest increase (3%) in fall rates among young children and a much larger increase (23%) in elderly fall rates between 2002 and 2010.

Although there was no significant increasing trend in fall rates over the study period, falls remain the most common mechanism of TBI, therefore reinforcing the need for injury prevention efforts focused on these high risk groups. Interventions targeting children include improved helmet laws and child safety equipment, safer sports practices, and increased TBI awareness [9–11]. Macpherson and Spinks [26] conducted a systematic review to assess the effects of bicycle helmet legislation on uptake of helmet use and head injury prevention among children. This review found evidence supporting the use of helmet legislation for increasing helmet use and decreasing head injury rates. In addition, several Cochrane reviews have examined the effectiveness of falls interventions among the elderly in both community and hospital settings. Gillespie et al. [27] reported that exercise programs and home safety interventions reduced fall rates and risk of falling among community-dwelling elderly populations, but multifactorial interventions based on individual risk assessments did not significantly reduce the risk of falling. Meanwhile, Cameron et al. [28] studied elderly populations in care facilities and hospitals and found evidence suggesting that multifactorial interventions including exercise programs, medication review, assistive technology, and educational programs may reduce the risk of falling in hospital.

Decreased rates of MVC and bicyclist-related injuries among children and young adults are attributable to successful injury prevention or health policy changes. In 1994, Graduated Driver Licensing (GDL) was introduced in Ontario requiring new drivers to pass through two stages of licensing. Additional restrictions have been added over time, including enhanced passenger restrictions in 2005 and a zero blood-alcohol concentration limit for drivers under age 22 in 2009 [23]. A recent Cochrane review [29] showed that GDL programs are effective in reducing crash rates, particularly among young drivers, with a 15.5% (range 8 to 27%) median decrease in crash rates among 16 year-old drivers. Stricter federal impaired driving laws and increased enforcement over the study period could have also contributed to reduced MVC rates [30]. In addition, decreases in bicyclist-related injuries have been documented after the introduction of mandatory helmet laws and helmet education campaigns [10, 26]. However, adolescents are less likely to comply with helmet use compared to younger children, and therefore remain a key population for focused educational efforts and health policy interventions [26, 31].

Our study highlighted trends towards increasing comorbidity, length of stay, and use of ambulatory transport among TBI patients treated in the ED. Our findings are consistent with previous studies that have shown an increase in the rate of severe TBI treated in EDs [6, 21]. These trends may reflect changes in medical practice which have shifted treatment of milder TBI to non-ambulatory care settings such as physician offices. A more complete picture of the healthcare burden of TBI could be obtained by linking administrative data from hospital and emergency department databases. These findings suggestive of increased injury severity may also reflect the increased reliance on CT scanning and advancements in imaging and trauma care, which may have improved the detection of intracranial injuries and management of critically injured patients. In addition, changes in ICD coding practices may have led to more frequent assignment of codes associated with increased severity.

To our knowledge, only one study has examined a variety of factors influencing hospital admission following TBI. McCarthy et al. [13] studied statewide epidemiological patterns of TBI among children (ages 0–19) in Maryland, and found that male sex, TBI severity and presence of major associated injuries, and transportation-related TBI were significantly associated with increased risk of hospitalization. Others studies have also reported a link between TBI-related hospitalization and increased comorbidity, injury severity, and mechanism of injury [2, 9]. These results are consistent with our findings that male sex, multiple comorbidities, MVCs, and ambulatory transport (a proxy for injury severity) were independent predictors of hospital admission following TBI.

This study was based on administrative NACRS data which may not capture certain demographics at risk for TBI such as prisoners or Aboriginal people living on federal reserves. The dataset also does not capture milder injuries treated in non-ambulatory outpatient care settings such as physician offices. In addition, our data is subject to potential miscoding, particularly given the large proportion of ED visits (49%) coded as “other unspecified head injuries” (S09.7-S09.9), which may represent other TBI or non-TBI diagnoses. Another ongoing issue is the lack of a standardized ICD-10 definition for TBI morbidity in the literature. Previous work [32] has demonstrated significant variation in TBI rates depending on the chosen ICD-10 definition, highlighting the need for a standardized definition to generate meaningful comparisons across studies. Furthermore, the lack of data on traditional severity measures such as GCS and ISS indicates the need for more consistent reporting of these measures in population-based datasets. This study likely underestimates the true healthcare burden of TBI, as studies show that at least 39% of TBI remain undiagnosed [3, 33]. Future studies are needed to link hospital, emergency department, and physician office databases to obtain a more comprehensive scope of the burden of TBI. Additional research is also warranted to assess long-term outcomes of TBI patients who are treated and released from the ED.

## Conclusions

In this population-based study of emergency department visits for TBI over an eight-year period, we found that ED visits for TBI are increasing in length of stay and use of ambulatory transport, and involve older populations with more complex comorbidities. While TBI rates are stable or declining among the vulnerable groups highlighted in this study, these populations remain key targets for focused injury prevention and enhanced surveillance. Clinicians in the ED setting should be cognizant of factors associated with hospital admission following TBI, including male sex, advanced age, multiple comorbidities, and ambulatory transport.
